# What Should I Eat? Dietary Recommendations for Patients with Inflammatory Bowel Disease

**DOI:** 10.3390/nu15040896

**Published:** 2023-02-10

**Authors:** Srishti Saha, Neha Patel

**Affiliations:** 1 Department of Internal Medicine, University of Texas Southwestern Medical Center, Dallas, TX 75390, USA; 2 Division of Digestive and Liver Diseases, University of Texas Southwestern Medical Center, Dallas, TX 75390, USA

**Keywords:** inflammatory bowel disease, diet, nutrition, micronutrient, additive, Crohn’s disease, ulcerative colitis, colitis, diarrhea

## Abstract

Inflammatory bowel disease (IBD) is a chronic disorder thought to be caused by enteric inflammation in a genetically susceptible host. Although the pathogenesis of IBD is largely unknown, it is widely accepted that dietary components play an important role. Human and animal-based studies have explored the role of various dietary components such as meat, artificial sweeteners and food additives in causing enteric inflammation. Several diets have also been studied in patients with IBD, specifically their role in the induction or maintenance of remission. The most well-studied of these include exclusive enteral nutrition and specific carbohydrate diet. A diet low in FODMAPs (fermentable oligosaccharides, disaccharides, monosaccharides and polyols), typically prescribed for patients with irritable bowel syndrome, has also been studied in a specific subgroup of patients with IBD. In this review, we describe the current evidence on how various dietary components can induce enteric and colonic inflammation, and the clinical–epidemiological evidence exploring their role in predisposing to or protecting against the development of IBD. We also discuss several special diets and how they affect clinical outcomes in IBD patients. Based on the available evidence, we provide guidance for patients and clinicians managing IBD regarding the best practice in dietary modifications.

## 1. Introduction

Inflammatory bowel disease (IBD) is a chronic inflammatory condition affecting the gastrointestinal system, the burden of which has been increasing globally [1,2,3]. In a systematic review of studies from 2010 to 2016, the incidence ranged from 0 to 29.3 per 100,000 person-years for Crohn’s disease (CD) and from 0.19 to 57.9 per 100,000 person-years for ulcerative colitis (UC), with wide variability in different regions [2]. The majority of studies report increasing incidence of both CD and UC in newly industrialized nations, but stable or decreasing incidence in North America and Europe [2]. Despite the variation in the time trends in incidence, the prevalence of IBD has been increasing, as was seen in a study using data from the Global Burden of Diseases, Injuries and Risk Factors Study (1990–2017), with the highest prevalence in North America [3]. 

IBD is characterized by inflammation in the gut resulting from an interplay between genetic and environmental factors. Of the various environmental factors, diet is considered to be a vital player in the pathogenesis of IBD, and unsurprisingly, both patients and physicians consider diet to be a vital component of the management of IBD. In a survey of patients recently diagnosed with IBD, over 70% thought that information on dietary measures and recommended nutritional supplements was important, and over 70% thought they did not receive adequate information on these issues [4]. Interestingly, less than half of gastroenterologists thought they had adequate information about dietary needs of patients with IBD, thus underlining the unfulfilled need for accurate information [5]. In this narrative review, we explore the evidence behind the role of commonly studied dietary components and specific diets in the management of patients with IBD. We highlight the strengths and limitations of the current evidence and provide a framework for clinicians to counsel patients regarding their diet.

## 2. Dietary Components and Food Groups

Dietary components such as red meat, various fats and food additives have been implicated in the pathogenesis of IBD, while others such as dietary fiber have been thought to be protective [6,7]. The mechanism behind these observed associations is via the disruption of the gut barrier, which propagates gut inflammation. In a systematic review including case-control and cohort studies, there was an increased risk of developing UC and CD with high intake of meat [8]. High meat consumption leads to a decrease in short-chain fatty acid (SCFA) oxidation, which drives breakdown of the mucus lining of the gut, thus thinning the gut barrier. Disruption of the gut mucosal barrier, an important natural protective mechanism, leads to increased permeability to enteric pathogens, which can increase gut inflammation. 

Considering the observed associations of high meat intake and the risk of developing IBD, investigators sought to determine if decreasing meat intake could affect clinical outcomes in established IBD. A randomized controlled trial (RCT) was conducted that looked at the effect of meat consumption on symptoms in patients with CD [9]. There was no difference in time to symptomatic relapse between patients who had low (less than one serving a month) vs. high (two or more servings a week) meat consumption, and a similar proportion of patients in either group suffered from a relapse (62% in the high-meat group vs. 42% in the low-meat group). Even though the diets in the two groups were not controlled, comparison of dietary patterns did not reveal any significant differences in nutrients except for mono-unsaturated fatty acids, which was higher in the high-meat-consumption group. Thus, while decreasing meat consumption may be helpful in mitigating the risk of IBD, we do not have sufficient evidence that lower meat intake improves outcomes in established IBD.

There has been interest in the association of dietary fats and the risk of IBD. Western diets often have a high quantity of omega-6-fatty acids such as arachidonic acid and linoleic acid, and a low quantity of omega-3-fatty acids such as docosahexaenoic acid and eicosapentaenoic acid. Omega-6-fatty acids have a pro-inflammatory effect, which propagates the damage caused by a disrupted gut barrier. Their pro-inflammatory action is mediated by (1) gut dysbiosis, which enables a microenvironment enriching pathogenic bacteria, (2) upregulation of pro-inflammatory genes and (3) a decrease in the proportion of bile acids ursodeoxycholic acid and deoxycholic acid [10,11]. In a systematic review, some authors noted a high risk of developing CD with saturated fats, total polyunsaturated fatty acids (PUFAs) and omega-6-fatty acids [8]. Similarly, there was a high risk of developing UC with the intake of total fats, total PUFAs and omega-6-fatty acids [8]. 

In contrast to high dietary fat and meat consumption, high dietary fiber and fruit intake decreases the risk of developing CD, but not UC [6,8]. This decreased risk is because dietary fiber provides the SCFAs necessary for nourishing the gut microbiome and enhances the gut epithelial barrier [8]. The effect of dietary fat and fiber modification in IBD patients was studied in a recently published RCT. In this study, 17 patients with UC (mild disease or in remission) were given a catered low-fat high-fiber diet and an improved standard American diet for four weeks each, using a cross-over study design [12]. Both diets improved quality of life, and the low-fat high-fiber diet also decreased intestinal dysbiosis and markers of gut inflammation. In contrast, several RCTs found that there was no benefit of omega-3-fatty acid supplementation for maintaining remission in either UC or CD [13,14,15]. The caveat to these studies is that there is no data regarding the baseline dietary patterns in the two groups, which can modify the efficacy of dietary intervention. 

Based on the current evidence, a guidance statement was released, in which the authors thought it prudent to increase the dietary intake of fruits and vegetables in CD (with the exception of stricturing disease) [16]. The evidence in UC was not enough to make specific recommendations. They also recommended reduced intake of processed or red meats in UC. Finally, it was recommended that the intake of dietary trans- or saturated fats should be reduced, and the intake of dietary omega-3-fatty acids should be increased. 

### 2.1. Sweeteners, Additives and Emulsifiers

Epidemiological studies have implicated an excess consumption of sugar or artificial sweeteners with higher risk of IBD, although no clinical studies have assessed whether limiting these dietary components is beneficial for inducing or maintaining remission [17,18,19]. Several additives and emulsifiers have been implicated in worsening of IBD. Carrageenan is a high-molecular-weight polysaccharide used as a thickener, gelling agent, emulsifier and stabilizer in many processed foods, especially reduced-fat products, such as ice cream, soymilk and yogurt. It is a pro-inflammatory molecule that induces innate immunity and has been found to induce colitis in animal models [20,21,22,23]. The role of a carrageenan-containing diet in inducing relapse in inactive UC was studied in a small randomized controlled trial (RCT) in which three out of five patients on a carrageenan-supplemented diet relapsed by 52 weeks compared to none of the seven in the placebo group (*p* = 0.046); however, no data regarding baseline intake of nutrients or food groups was provided [24]. Thus, it may be prudent to limit the intake of additives such as carrageenan in the diet, although data to support such a recommendation are limited.

### 2.2. Curcumin Supplementation

Several studies have explored the role of curcumin in inducing or maintaining remission in patients with IBD. Curcumin is a component in turmeric, a spice used in Indian cuisine, and has been used in ayurvedic and traditional Chinese medicine for treating inflammatory conditions. It is known to have anti-oxidant and anti-inflammatory properties in gut epithelial cells. It acts via inhibition of IFN-γ signaling, which inhibits epithelial cell migration, affects gut barrier function and impairs wound healing [25]. Moreover, it can impair neutrophil migration across the gut epithelium, a process that impairs gut barrier function and propagates gut inflammation and tissue destruction [26]. 

In a multi-center randomized placebo-controlled trial including 50 patients with mild–moderate UC, curcumin (3 g/day) was added to oral and rectal mesalamine therapy [27]. The addition of curcumin (*n* = 14) led to increased rates of clinical remission (54% vs. 0%, *p* = 0.01), endoscopic remission (38% vs. 0%, *p* = 0.04) and clinical response (65% vs. 13%, *p* < 0.01). In another RCT including 89 patients, the rates of relapse were compared between patients receiving curcumin (2 g/day) vs. those of placebo in addition to mesalamine and sulfasalazine for quiescent UC [28]. The rates of relapse were significantly lower in the curcumin group (5% vs. 21%, *p* = 0.04) at 6 months of follow up. In a single-center pilot RCT (*n* = 45) including patients with mild–moderate distal UC, daily curcumin enema plus 5-ASA was superior to placebo plus 5-ASA in inducing clinical remission and clinical response at 8 weeks, although this was only seen in a per-protocol analysis [29]. One RCT studied the efficacy of a highly bioavailable synthetic curcumin derivative Theracurmin^®^ in patients with mild–moderate Crohn’s disease (*n* = 30; 20 in intervention arm, 10 in placebo arm) [30]. In this study, the study drug was significantly better at inducing clinical remission at 12 weeks compared to placebo (40% vs. 0%, *p* = 0.02) and was better at anal healing (63% vs. 0% at 8 weeks, *p* = 0.02). Notably, patients in both groups were on other treatments for CD (steroids, immunomodulators, biologics and 5-ASA compounds), although the distributions of each of these therapies were similar in both arms. A systematic review and meta-analysis was conducted to study the role of oral curcumin in inducing remission in patients with UC. Only three RCTs met the criteria for inclusion, and the results were inconsistent, dependent on the method of analysis used. The study authors concluded that the data do not support the use of curcumin in UC patients. 

The benefit of using curcumin lies in its good safety and tolerability profile. However, evidence on the use of curcumin is limited by several factors: first, there are only a limited number of RCTs that have studied this supplement. Second, all the studies have a limited sample size. Third, there is heterogeneity in study design (extent of disease, endpoints studied, duration of follow up, mode and dose of administration of curcumin). Given these limitations, it may be reasonable to add curcumin as an adjunct therapy in patients with mild–moderate UC to induce or maintain remission. It could be particularly useful in patients who would prefer to use natural/herbal products or are unable to tolerate the usual medical therapies. However, more high-quality data are needed to support this recommendation and to implement it in everyday practice. Given the scarcity of studies done on CD, we would recommend against its use in patients with CD. Importantly, given the promising data in most studies done to date, larger randomized controlled trials should be done to provide high-quality evidence regarding its use in both CD and UC patients.

## 3. Special Diets 

Several different diets have been tried and tested in patients with IBD, with the goal of inducing remission or reducing disease flares (Table 1). 

### 3.1. Exclusive Enteral Nutrition (EEN)

Exclusive enteral nutrition (EEN) was traditionally used for nutritional support in patients with CD awaiting surgery. Over the years, data have accumulated regarding the use of EEN in induction and maintenance of remission of CD. EEN is classified based on the dietary composition—elemental diets comprise entirely amino-acids and are antigen-free; semi-elemental diets comprise oligopeptides; polymeric diets comprise whole protein derived from foods such as milk and eggs. The role of EEN is best known in pediatric CD and was recommended as the first-line therapy for induction of remission in luminal CD by the European Crohn’s and Colitis Organization [31]. In a Cochrane systematic review and meta-analysis of RCTs, investigators found that elemental and non-elemental diets were similar in inducing remission in active CD [32]. When compared to steroids, EEN had similar efficacy overall in inducing remission in CD. Interestingly, on subgroup analyses, the efficacy of steroids was superior to EEN in adults (73% vs. 45%) but not in the pediatric age group (61% vs. 83%, respectively). However, the results were of very low quality and were affected by type of analysis (intention-to-treat vs. per-protocol) and risk of bias of studies [32]. In another systematic review of clinical trials, investigators explored the role of enteral nutrition in maintenance of remission in CD [40]. However, only four studies met the inclusion criteria, and no conclusions could be drawn from the available evidence. 

One of the challenges with EEN is adherence and tolerability, particularly in adults. To address this issue, alternate diets have been devised, such as the CD-TREAT diet and the CD exclusion diet (CDED) in concert with partial enteral nutrition (PEN). The CD-TREAT diet is a personalized diet that recreates EEN by excluding certain components such as alcohol, gluten and lactose and matching other components such as vitamins, fiber, minerals and micronutrients. The CDED is a whole-foods-based diet that excludes certain components such as wheat, animal fat, dairy, packaged or canned goods, artificial sweeteners and other additives that are known to induce gut inflammation and cause dysbiosis. Clinical studies have shown both diets to be equally efficacious and better tolerated than EEN [10,41]. A recent systematic review of prospective controlled trials found potential benefit of PEN in induction and maintenance of remission in CD [42]. 

Putting the evidence together, the role of EEN may be limited to certain populations, such as pediatric IBD or adult CD patients who are hospitalized for an acute flare and consequently can be closely monitored. In adults, EEN maybe useful as an adjunct to standard-of-care therapies. The lack of efficacy of this diet, particularly in the adult population, is at least partially explained by difficulty in adherence. Alternate exclusion diets such as CD-TREAT and CDED + PEN are likely more “palatable” and should be explored. 

### 3.2. Specific Carbohydrate Diet

The specific carbohydrate diet (SCD) is a diet that eliminates complex carbohydrates and replaces them with simple carbohydrates such as monosaccharides. Undigested complex carbohydrates remain in the gut for prolonged periods of time, providing substrate for pro-inflammatory bacterial species. This propagates mucosal thinning and increases gut permeability. Thus, the hypothesis is that SCD would reduce gut inflammation and improve gut barrier function. The diet allows the inclusion of unprocessed meats, simple carbohydrates (monosaccharides), all fats and oils, aged cheeses, lactose-free yogurt and fresh fruits and vegetables except for some starchy vegetables. However, it prohibits milk products, soft cheeses, sweeteners except honey and grains.

Current data on the SCD in IBD are mostly in the pediatric population. In a small pilot study, three types of diets were tested in pediatric CD patients—SCD, modified SCD (SCD with oats, rice), whole foods [34]. The study included a total of ten patients, who had relatively mild disease at baseline. After 12 weeks of dietary modification, CRP and disease activity decreased in all patients. In another small clinical trial, nine pediatric patients with CD had decreased disease activity scores after 12–52 weeks of therapy with the SCD [33]. In a small retrospective study with 20 pediatric CD and six pediatric UC patients, there was a reduction in mean disease activity score by 24 points in the CD group and by ten points in the UC group after six months on the SCD [43]. A prospective study called PRODUCE (NCT03301311) is currently underway and will shed light on the efficacy of strict SCD vs. modified SCD in pediatric IBD.

The largest study on the SCD to date (DINE-CD) is an RCT in adults with CD, wherein the SCD was compared to the Mediterranean diet as a treatment for patients with mild–moderate disease [35]. Six weeks after randomization of the 196 included patients, there were no differences in clinical remission, fecal calprotectin response or CRP response between the two groups, though over 40% of patients in both groups achieved remission. The high remission rates are promising, though the effect of a particular diet vs. the use of fresh ingredients is unclear. Despite the “negative” results from the trial, it should be noted that the response rates were high in both groups, which suggests that either diet may be superior to a standard western diet for patients with mild–moderate CD. 

In another study conducted in adults and children with either UC or CD, patients were administered surveys to assess the efficacy of the SCD [44]. The proportion of patients reporting symptoms (abdominal pain, diarrhea, activity limitations, weight loss) decreased from 58 to 81% pre-diet to <10% after 12 months of the SCD diet [44]. The perception of remission went up from 4% pre-diet to 42% at 6–12 months post-diet. The study was limited by recall bias, response bias, unclear dietary compliance and quality (less than half of the patients were on a strict SCD diet without modifications). 

To conclude, the SCD is a restrictive diet with unclear benefits in both the adult and pediatric populations. Based on the DINE-CD study, some dietary restriction is helpful, and either the SCD or the Mediterranean diet may be tried as an option in adults (Table 1). However, given the ease, availability and non-IBD benefits, the Mediterranean diet may be preferred as the first choice. Much more high-quality data, including data comparing the SCD to standard western diet, are needed in order to establish the role of the SCD in IBD.

### 3.3. Low FODMAP Diet

Approximately 39% of patients with IBD report symptoms of irritable bowel syndrome (IBS) [45]. The low FODMAP diet (low in fermentable oligosaccharides, disaccharides, monosaccharides and polyols) has been traditionally used in patients with IBS and functional GI disorders. It eliminates certain foods that are poorly digested and highly fermented in the gut, causing flatulence and GI distress. The low FODMAP diet is typically undertaken in three phases: elimination (intended as a short-term phase), reintroduction and maintenance. 

Several studies have explored the role of the low FODMAP diet in IBD. An open-label trial compared the low FODMAP diet to a standard diet in 78 IBD patients (in remission or with mild–moderate disease) with concurrent IBS symptoms [37]. They found that patients on the low FODMAP diet had a better response in IBS symptoms (81% vs. 46%) and better quality of life. In a small randomized cross-over trial of low vs. typical FODMAP diet in patients with quiescent CD, there was an improvement in symptoms with the low FODMAP diet but no difference in fecal calprotectin levels [36]. In another RCT of 52 patients with quiescent IBD, there was a reduction in symptom severity scores but not overall IBS symptoms score, and no difference in inflammatory markers or IBD disease activity score four weeks after the low FODMAP diet [46]. In a retrospective study including 72 patients with IBD on the low FODMAP diet, over half reported improvement in abdominal pain, diarrhea and bloating after three months of the diet [47]. However, no assessment of IBD activity either at baseline or at follow up was available. 

To summarize, studies of the low FODMAP diet have shown improvement in symptoms in IBD patients with low or no disease activity, particularly in those with concurrent IBS (Table 1). However, there is no evidence to date that explores the role of the low FODMAP diet in induction or maintenance of remission in active IBD. Hence, there may be a role of this diet in IBD patients with persistent symptoms despite clinical remission, likely due to its effect on GI symptoms rather than gut inflammation. More data are needed to explore the role of the low FODMAP diet in active IBD.

### 3.4. Plant-Based Diets 

High meat consumption and low consumption of fresh fruits, vegetables and fiber have been associated with maladaptive changes in the gut leading to a pro-inflammatory state, as has been discussed previously in this article. Due to these changes, there has been an interest in plant-based diets (rich in fruits, vegetables, nuts, legumes and grains) as a therapeutic intervention for IBD (Table 1). A semi-vegetarian diet (SVD) is similar to plant-based diets but allows milk, eggs and fish every week and meat every two weeks. A vegetarian diet, on the other hand, completely eliminates meat products. In a single-center study, 22 patients with CD who had achieved remission medically or surgically were continued on SVD if they were on it during hospitalization. Remission was maintained in 94% patients in the SVD group compared to 33% in the control group [38]. However, there was no adjustment for other factors that could have affected relapse rates such as level of inflammation or choice of induction and maintenance therapy. In an observational study of 1254 patients with IBD, there was no benefit overall with a vegetarian diet (disease activity, complication rates), though patients in the CD subgroup had lower complication rates with the vegetarian as compared to those with the meat-containing diet (42% vs. 60%) [48]. Of note, these were not adjusted for other risk factors of disease-related complications.

Ultimately, while there is biological plausibility to using plant-based diets, more high-quality evidence is needed to better understand their role in the treatment of IBD.

### 3.5. Anti-Inflammatory Diet

The IBD anti-inflammatory diet (IBD-AID) was created to reduce inflammation in the gut and consequently reduce the frequency and severity of IBD flares and maintain remission. It is based on the theory that certain carbohydrates provide a substrate for pro-inflammatory pathogenic microbes to flourish in the gut lumen, thereby initiating and maintaining a cascade of gut inflammation [39]. The IBD-AID has five components: (1) modification of carbohydrate intake, (2) ingestion of pre- and probiotics, (3) modification in fat intake, (4) review of dietary intake, food intolerance and missing nutrients and (5) modification of food textures to improve nutrient absorption and reduce intact dietary fiber [39]. The diet consists of lean meats, poultry, fish, specific carbohydrate elimination (highly processed or refined carbohydrates, lactose), inclusion of pre- and probiotics (onion, leeks, fermented vegetables, fresh cultured yogurt, kefir, miso, natural soluble prebiotics such as banana, oats and flax meal), modification of fats (vegetable oils, omega-3 oils, reduced total and saturated fats), and includes a limited array of dairy products (yogurt, aged cheeses). Additionally, the diet begins with soft, pureed textures and gradually progresses to more solid textures to slowly improve tolerance.

To study this diet, investigators offered the diet to 40 consecutive patients with IBD, of which only 24 followed it [39]. They then retrospectively reviewed the records of the patients (only 11 had complete data available). Of these 11 patients, eight had CD and three had UC. All patients showed improvement in disease severity indices from baseline to 1–4 weeks post-dietary modification, and all of them were able to reduce at least one IBD-related medication. However, the study was small, it was a case-series, and less than half of the patients who were offered the diet had complete data available for analysis. 

A recently published randomized controlled trial evaluated the effect of fecal microbiota transplantation (FMT) in conjunction with an anti-inflammatory diet in induction and maintenance of remission in 66 patients with mild–moderate UC [49]. In this study, compared to those with standard medical therapy, the patients in the FMT-AID arm had significantly higher rates of remission at 8 weeks (60% vs. 32%, *p* = 0.02) that was maintained with continuation of AID after the attainment of remission (25% vs. 0%, *p* = 0.007). While this study was not designed to study AID alone, it provides indirect evidence that AID may have helped in maintaining remission after FMT. However, whether it was the long-term effect of FMT alone or the combination of FMT and AID that helped maintain remission cannot be determined. Moreover, whether AID was helpful in inducing remission cannot be determined with this study given that the patients concomitantly received FMT.

Several clinical trials studying the role of AID are ongoing (clinicaltrials.gov accessed on 3 February 2023: NCT04431700, NCT04913467, NCT02093780 and NCT02357537) and will provide more data regarding its efficacy in IBD. Until such high-level evidence is available, clinicians should not recommend it to their patients.

## 4. Conclusions

Despite the perceived importance of diet in the pathogenesis and management of IBD, limited high-quality data exists to provide strong recommendations. Based on the evidence to date, patients with IBD may benefit from limiting the consumption of trans- and saturated fats, omega-6-fatty acids, artificial sweeteners and additives such as carrageenan. Observational data support the benefits of a diet rich in fruits, vegetables and omega-3-fatty acids. Among the specific diets, exclusive enteral nutrition may be beneficial in specific subgroups of patients with Crohn’s disease, and the role of diets such as the Crohn’s disease exclusion diet, anti-inflammatory diet and specific carbohydrate diet needs to be explored further. Finally, patients with IBD and persistent GI symptoms may benefit from a low FODMAP diet. 

## Figures and Tables

**Table 1:** Summary of evidence on commonly used diets in IBD and key take away points.

Diet	Components	Proposed Mechanisms	Key Inferences from Studies to Date
Exclusive enteral nutrition (EEN)	Exclusive formula-based liquid diet for 4–12 weeks (per oral or via NGT).	Exclusion of potentially “damaging foods”. Modulation of host immune response. Microbial changes. Gut barrier protection.	Possible role in pediatric CD [31]. No difference between elemental and non-elemental formulations in efficacy [32]. EEN is inferior to steroids in inducing remission in CD in adults [32].
Specific carbohydrate diet (SCD)	Monosaccharides (glucose, galactose, fructose), fresh fruits, vegetables, unprocessed meats, yogurts, nuts, hard cheeses. Avoidance of dairy products, complex carbohydrates, grains.	Decreasing growth of pro-inflammatory bacterial species.	Pilot studies with some indication of benefit in pediatric IBD (reduced disease activity scores) [33,34]. Limited data in adults. SCD is not different from the Mediterranean diet in inducing remission in adults with CD based on one clinical trial [35].
Low FODMAP diet	Restriction of fermentable oligosaccharides, disaccharides, monosaccharides and polyols.	Decreasing flatulence caused by high FODMAPs. Reducing gut inflammation. Protecting gut mucosal barrier.	Reduction in GI symptoms (bloating, abdominal pain, diarrhea) in quiescent or mild–moderate IBD [36,37].
Plant-based diets	Rich in fruits, vegetables, nuts, legumes, grains, soy. -In semi-vegetarian diet, milk, dairy, eggs and fish allowed weekly, meat once every two weeks	Eliminating the pro-inflammatory state induced by high meat and low fiber consumption	SVD may be of benefit in maintaining remission in CD, but data is not adequate to provide specific recommendations [38].
Anti-inflammatory diet	Includes plant-based proteins, lean meats, fish, vegetables, fruits, vegetable oils, prebiotics and probiotics. Avoidance of red meats, reduction of total and saturated fats, insoluble fiber. Implemented in four phases, with varied nutrients and food textures in each phase.	Elimination of pro-inflammatory food components. Reducing gut microbial dysbiosis by reducing substrate for the growth of pathogenic bacteria.	Small case series showed improvement in disease activity scores (included both CD and UC patients) [39]. No randomized controlled trial evaluating its efficacy as a standalone intervention.

CD, Crohn’s disease; EEN, exclusive enteral nutrition; FODMAPs, fermentable oligosaccharides, disaccharides, monosaccharides, and polyols; IBD, inflammatory bowel disease; NGT, nasogastric tube; SCD, specific carbohydrate diet; SVD, semi-vegetarian diet.

## Data Availability

No new data were created as this was a review of studies.
